# Topical Chinese patent medicines for chronic musculoskeletal pain: systematic review and trial sequential analysis

**DOI:** 10.1186/s12891-023-07072-8

**Published:** 2023-12-20

**Authors:** Kaiqiang Tang, Jigao Sun, Yawei Dong, Zelu Zheng, Rongtian Wang, Na Lin, Weiheng Chen

**Affiliations:** 1 The Third Affiliated Hospital of Beijing University of Chinese Medicine, No. 51 Anwai Xiaoguanjie, Chaoyang District, Beijing, 100029 PR China; 2 Department of Orthopedics, Dongfang Hospital Beijing University of Chinese Medicine, Beijing, China; 3 Institute of Chinese Materia Medica, China Academy of Chinese Medical Sciences, Beijing, China

**Keywords:** Topical Chinese patent medicine, Traditional Chinese medicine, Chronic musculoskeletal pain, Trial sequential analysis, Systematic review

## Abstract

**Purpose:**

Chronic musculoskeletal pain (CMP) is defined as persistent or recurrent pain that occurs in the joints, musculo-soft tissue, spine or bones for more than three months and is not completely curable. Although topical Chinese patent medicine (CPM) is the most extensively utilized medication in Asia and is widely used for pain management, its efficacy remains controversial. This article presents a systematic review of clinical studies on the therapeutic properties of topical CPM for CMP patients to better inform clinical decision-making and provide additional and safer treatment options for patients with CMP.

**Method:**

We performed a comprehensive search on PubMed, Cochrane Library, web of science and Chinese databases (CNKI and WanFang data) from 2010 to 2022. In all the studies, knee osteoarthritis, cervical spondylosis, low back pain, and periarthritis of shoulder met the International Pain Association definition of chronic musculoskeletal pain. We included only randomized controlled trials (RCTs) using topical CPM primarily for chronic musculoskeletal pain in adults. To determine the effect of topical CPM on clinical symptoms, we extracted the Visual Analog Scale (VAS, range 0–10) and the Western Ontario and McMaster Universities Arthritis Index pain scores (WOMAC pain, range 0–20), in which the lower the score, the better the results. We also accepted the comprehensive outcome criteria developed by the Chinese National Institute of Rheumatology as an endpoint (total effectiveness rate, range 0–100%, higher score = better outcome), which assesses the overall pain, physical function and wellness. Finally, trial sequential analysis of VAS pain score and total effectiveness rate was performed using TSA software.

**Results:**

Twenty-six randomized controlled trials (*n* = 3180 participants) compared topical CPM with oral Nonsteroidal Anti-inflammatory Drugs (NSAIDs) (*n* = 15), topical NSAIDs (*n* = 9), physiotherapy (*n* = 5), exercise therapy (*n* = 4), and intra-articular Sodium hyaluronate injection (*n* = 2). Sixteen studies found that topical CPM was statistically significant in improving CMP pain (measured by VAS pain and Womac pain scores)(*p* < 0.05), and 12 studies found topical CPMs to be more clinically effective (assessed by ≥ 30% reduction in symptom severity) in treating patients with CMP (*p* < 0.05). Trial sequential analysis indicates that the current available evidence is robust, and further studies cannot reverse this result. In most of the studies, randomisation, allocation concealment and blinding were not sufficiently described, and no placebo-controlled trials were identified.

**Conclusion:**

Most studies showed superior analgesic effects of topical CPM over various control treatments, suggesting that topical CPM may be effective for CMP and is an additional, safe and reasonable treatment option. These reported benefits should be validated in higher-quality RCTs.

**Supplementary Information:**

The online version contains supplementary material available at 10.1186/s12891-023-07072-8.

## Introduction

Chronic musculoskeletal pain (CMP) is defined as persistent or recurrent pain that occurs in the joints, musculo-soft tissue, spine, or bones for greater than three months [1]. Depending on the site and mechanism of onset, CMP can be classified as chronic primary or secondary joint-derived pain, chronic musculo-soft tissue-derived pain, spine-derived pain, or bone-derived pain, such as knee osteoarthritis (OA), lower back pain (LBP), neck pain (NP), and periarthritis of shoulder (FS),etc. [2–4]. CMP has become the leading cause of disability worldwide in the last two decades, with 20–33% of the global population suffering from musculoskeletal disorders [5], and its visit rate is second highest among chronic pain patients [6]. According to 2021 data, the number of people with CMP in Latin America is 21.8 percent, and the prevalence may continue to climb [7]. Chronic pain can have a negative effect on a person's quality of life, and in severe cases, it may result in functional incapacity. Recent clinical practice guidelines, such as the *OARSI guidelines for the nonsurgical management of knee, hip, and polyarticular osteoarthritis*, strongly recommend topical medication for knee OA [8], and clinical treatment guidelines for LBP similarly recommend certain complementary and alternative medicine (CAM) interventions. Traditional Chinese medicine (TCM)-related treatments are used for patients with LBP, which has also been recognized [9, 10].

Topical Chinese patent medicine (CPM) has been widely utilized in China as an important treatment modality for CMP with some efficacy. Topical CPM is a Chinese medicinal preparation manufactured from Chinese herbs and processed according to precise prescriptions and manufacturing procedures guided by TCM philosophy, which is founded on the premise of disease prevention and treatment. The earliest reference of topical CPM is in the "Fifty-two Prescriptions for Diseases" (202 B.C.-9 A.D.), the earliest Chinese prescription book, which contains over 30 different types of topical CPM, most of which are used to treat traumatic injuries to tendons and bones. Local treatment of osteoarthritis with curative effects was also documented in the earliest published medical work in China, the Yellow Emperor's Classic of Internal Medicine (475 B.C. -221 B.C.). In recent years, with the development of TCM preparations, many classical Chinese medicines have been developed into proprietary TCM preparations after being approved by the relevant Chinese state authorities. Notably, topical CPM is currently widely utilized in Asian countries and is accepted by physicians and patients, particularly for the treatment of knee OA and other chronic musculoskeletal disorders. The most recent Chinese domestic guidelines for the treatment of osteoarthritis also encourage the use of topical CPM, stating that it possesses analgesic, anti-inflammatory, and circulatory improvement properties [11].

However, the evidence on the efficacy of topical CPM in relieving pain is inconsistent across a spectrum of common musculoskeletal pain presentations. Given the unresolved controversy regarding the clinical effectiveness of topical CPM for CMP, we conducted the first systematic evaluation of topical CPM for CMP, incorporating nearly ten years of literature and systematic reviews in English and Chinese from 2010 to 2021, with the aim of providing normative therapeutic evidence to support determining the clinical efficacy of topical CPM and better informing clinical practice.

## Methods

### Protocol and registration

This study was designed according to the recommendations of the Cochrane Handbook for Systematic Reviews of Interventions and the Preferred Reporting Items for Systematic Reviews and Meta-Analyses (PRISMA) statement. The study has also been registered on PROSPERO (CRD42022309212).

### Search strategy

To determine the therapeutic effect of topical CPM on CMP, we conducted a comprehensive search in PubMed, Cochrane Library, EMBASE, Web of Science, Chinese National Knowledge Infrastructure (CNKI), Wanfang data, and other databases from January 2010 to August 2022. Furthermore, we manually searched for publication records from the databases. The publication language was limited to Chinese and English. The search strategies employed both controlled vocabulary terms and keywords for CMP and topical CPM.

**Fig. 1:**
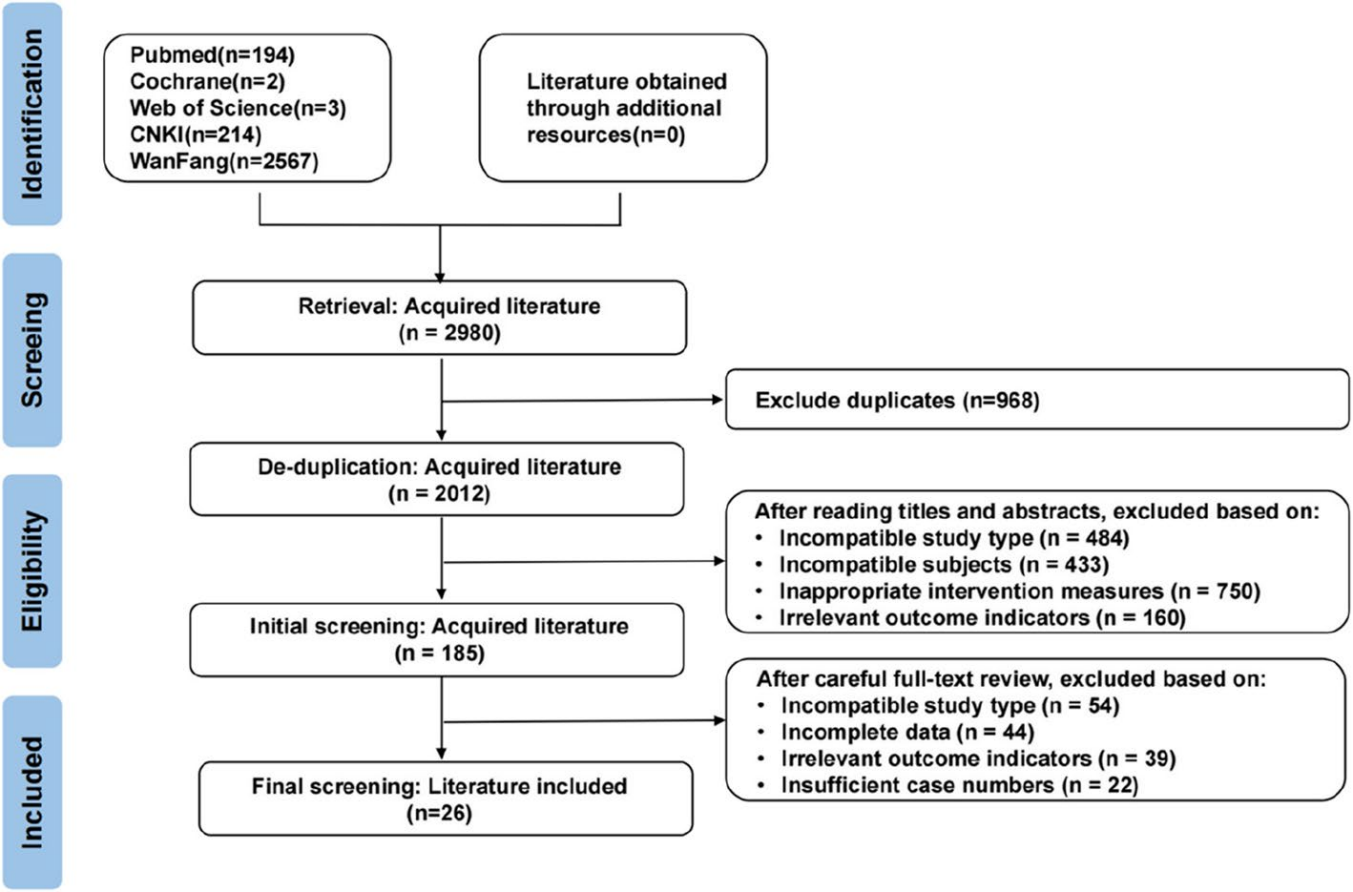
The flow of the literature search and publication selection process following PRISMA guidelines

According to the International Association for the Study of Pain (IASP) classification of chronic pain [1], we divided the search keywords into three categories based on the origin of CMP: (1) pain in the spine (including cervical spondylosis, lumbar sprain, low back pain, and lumbar disc herniation); (2) pain in joints (including OA, rheumatoid arthritis); and (3) pain in muscles or tendons (including fibromyalgia, carpal tunnel syndrome, soft tissue injuries, tennis elbow, rotator cuff injury, fasciitis, tenosynovitis, lumbar muscle strain,chronic ankle injury and calcaneodynia). The process of literature acquisition is shown in Fig. 1.

### Eligibility criteria

We included several randomized controlled trials (RCTs) that used topical CPM primarily for CMP. To meet the conditions of this study, the experimental group had to receive CPM therapies, and the control group had to be treated with only non-Chinese medicine interventions, such as NSAIDs, Voltaren Emulsion, or infrared physiotherapy. There was no restriction on the duration of interventions or the number of subjects in each group. We also selected some systemic reviews and meta-analyses.

### Study selection

Two researchers independently filtered all studies meeting the criteria. First, we excluded irrelevant articles according to their titles and abstracts. The full text of all potential literature was searched and filtered according to the research qualification criteria. Disagreements were resolved by consensus among team members. Due to the heterogeneity of interventions and comparisons, a meta-analysis was not performed. Disagreements were resolved by consensus among team members.

Pain level was the main outcome to evaluate the clinical efficacy of CPM in the treatment of CMP. In these studies, different scales, such as the visual analog scale (VAS), Western Ontario and McMaster Universities osteoarthritis index (WOMAC), were selected as outcomes of the pain level. These scales differ in their evaluation methods and applicable diseases. The VAS score ranges from 0 (no pain) to 10 (worst pain), where a higher score represents a worse outcome. The WOMAC score is widely used in the study of osteoarthritis. It consists of three aspects, joint pain, stiffness, and joint function, and contains 24 questions with a score of 0 ~ 4. The higher the score, the more serious the patient's condition is.

We also used the total effectiveness rate (TER) [12] to measure the effects of topical CPM on clinical symptoms. The total effectiveness rate is usually defined by the guidance principle of clinical study for new drugs in TCM and is calculated according to the number of patients in each of the following categories: (1) clinically cured: the main clinical manifestation score is reduced by 95%; (2) significant improvement: the main clinical manifestation score is reduced by 70% to 94%; (3) improvement: scores for major clinical manifestations are reduced by 30% to 69%; and (4) ineffective: scores for primary clinical manifestations are reduced by 30%.

### Data extraction

Data extraction was completed by one researcher and confirmed by at least one other researcher. The extraction contents mainly included basic information on the study characteristics; baseline information on the research object; type, duration, and frequency of the interventions; relevant data of the outcome indicators, etc. The total effectiveness rate was analyzed and compared by percentage, and the standardized mean difference (SMD) was used as the efficacy analysis statistic.

### Statistical analysis

The effect size for continuous variables was represented by the standardized mean difference (SMD), which indicates the trend of increase or decrease compared to the control group. This is intuitively presented in Table 1. The relevant calculations were performed using RevMan 5.4 software. Due to the heterogeneity of interventions and comparisons, a meta-analysis was not performed, thus we did not conduct sensitivity and subgroup analyses. However, based on the different sources of pain, we summarized and discussed the findings separately for different pain locations.

**Table 1:** 33 RCTs of topical Chinese patent medicine for chronic musculoskeletal pain

Author, year	N^a^	Age^b^	Topical CPM (Formula, dose)^c^	Control Intervention	Duration (wks)	Outcome Measures	Effect on Symptom (SMD or Percentage)	*P* value
**Disease name: Knee Osteoarthritis (ICD-11 Code: FA01.Z)**								
Hu, 2011 [41]	60	62	Xiaotongtie plaster, once/day, 24 h at a time	Celecoxib capsules, 200 mg, once/day	2	TER	Treatment effect: 90% vs. 86.6%↑	> 0.05
Xu, 2011 [16]	100	-	Xiaotongtie plaster, once/day, 24 h at a time	Meloxicam tablets, 7.5 mg, twice/day	1	VAS pain	-0.46↓	< 0.05
Guo, 2011 [15]	180	59	Xiaotongtie plaster, once/day, 24 h at a time	Diclofenac tablets, 75 mg, twice/day	2	TER	Treatment effect:↑ 91.9% vs. 78.0%	< 0.05
Wang, 2011 [42]	80	53	Xiaotongtie plaster, once/day, 6 h at a time	Diclofenac Tablets, 25 mg, twice/day	1	VAS pain	-1.35↓	< 0.01
Zhang, 2011 [43]	60	55	Xiaotongtie plaster, once/day, 24 h at a time	Diclofenac tablets, 75 mg, 1–2 times/day	4	TER	Treatment effect:↑ 93% vs.56%	< 0.01
Lv, 2011 [44]	160	60	Xiaotongtie plaster, once/day, 24 h at a time	Diclofenac tablets, 100 mg, once/day	1	VAS pain	-1.5↓	< 0.001
Lu, 2011 [45]	62	65	Xiaotongtie plaster, once/day, 24 h at a time	Diclofenac tablets, 25 mg, 3 times/day	1	TER	Treatment effect:↑ 86.67% vs. 84.38	> 0.05
Xin, 2011 [17]	200	65	Xiaotongtie plaster, once/day, 24 h at a time	Indomethacin Cuptalam plaster, once/day, for 24 h at a time	1	TER	Treatment effect:↑ 92% vs. 84%	< 0.05
Li, 2011 [46]	60	59	Xiaotongtie plaster, once/day, 24 h at a time	Voltaren emulsion, 4 times/day	1	VAS pain	-1.82↓	< 0.05
Wang, 2011 [47]	51	41	Xiaotongtie plaster, once/day, 12–24 h at a time	Voltaren emulsion, 2–4 times/day	1	TER	Treatment effect:↑ 100% vs. 79.2%	< 0.01
Li, 2019 [48]	80	52	Xiaotongtie plaster, once/day, 24 h at a time + herb fumigation	Diclofenac sodium sustained-release tablets, 75 mg, twice/day	8	WOMAC pain TER	-0.82↓ Treatment effect:↑ 92.50%vs. 72.50%	< 0.001 < 0.05
Shen, 2012 [18]	100	53	Fufang Nanxing Zhitong plaster, once/2 days, 24 h at a time	Local hot compress, 15 min, 3 times/day	1	VAS pain	-1.36↓	< 0.05
Son, 2012 [19]	108	53	Fufang Nanxing Zhitong plaster, once/2 days, 24 h at a time	Voltaren emulsion, 3 times/day	2	TER	Treatment effect:↑ 91.07% vs. 73.08%	< 0.05
Li, 2013 [49]	50	60	Fufang Nanxing Zhitong plaster, once/day, 8–12 h at a time	Sodium hyaluronate, once/5 days	4	VAS pain	-1.33↓	< 0.001
Chen, 2016 [50]	72	58	Tongluo Qutong plaster, once/day, no more than 12 h at a time	Diclofenac sodium, 3–4 times/day	2	VAS pain	-1.24↓	< 0.001
Qin, 2016 [21]	105	59	Tongluo Qutong plaster, once/day, for no more than 12 h at a time	Celecoxib tablets, 100 mg, twice/day	4	TER	Treatment effect:↑ 92.73 vs. 72.00	< 0.05
**Disease name: Cervical spondylosis (ICD-11 Code: FA8Z)**								
Son, 2016 [51]	80	42	Fufang Nanxing Zhitong Plaster, once/2 days, 24 h at a time	Wax therapy, twice/day, 30 min at a time	6	VAS pain	-0.75↓	< 0.05
Guo, 2017 [52]	240	32	Fufang Nanxing Zhitong Plaster, once/2 days, 24 h at a time	Infrared physiotherapy treatment, twice/day, 30 min at a time	6	VAS pain	-2.91↓	< 0.001
**Disease name: Low back pain (ICD-11 Code: ME84.2Z)**								
Li, 2011 [26]	400	40–70	Qingpeng Ointment, 10–20 g/day, + Xiaotongtie Plaster, once – 4 times/day	Waist Radiofrequency physical therapy + diclofenac tablets, once/day	-	VAS pain	-1.25↓	< 0.001
Zhou, 2011 [53]	100	16–72	Xiaotongtie Plaster, once/day, 24 h at a time	Diclofenac sodium sustained-release tablets, 50 mg, 3 times/day	2	VAS pain TER	-0.87↓ Treatment effect:↑ 82% vs. 56%	< 0.05
Wang, 2011 [54]	120	37	Xiaotongtie Plaster, once/day, 24 h at a time	Voltaren Emulsion, twice/day	2	VAS pain TER	-0.49↓ Treatment effect:↑ 92% vs. 79%	< 0.05 < 0.01
Son, 2013 [55]	114	43	Fufang Nanxing Zhitong Plaster, once/2 days, 24 h at a time	Capsaicin ointment, 0.5 g, 3 times/day	2	VAS pain TER	-0.36↓ Treatment effect:↑ 93.3% vs. 88.9%	< 0.05 > 0.05
Tian, 2013 [56]	200	43	Fufang Nanxing Zhitong Plaster, once/days + exercise therapy	Exercise therapy, 3 times/day, 10–20 reps per set	4	VAS pain	-5.29↓	< 0.01
**Disease name: Periarthritis of the shoulder (ICD-11 Code: FB53.0)**								
Yu, 2011 [57]	240	55	Xiaotongtie Plaster, once/day, 24 h at a time + massage	Dexamethasone 1 ml + prednisone 1 ml + Vitamin B12 1 ml, twice/week	3	TER	Treatment effect:↑ 94.17% vs. 81.78%	< 0.05
Zhou, 2016 [30]	60	54	Xiaotongtie Plaster, once/day, 24 h at a time	Functional exercise,half an hour each time, 3 times/day	4	TER	Treatment effect:↑ 76.7% vs. 63%	< 0.05
Tang, 2020 [22]	98	50	Xiaotongtie Plaster, once—twice/day, 4–6 h at a time + moxibustion, once/day, 5 min at a time	Meloxicam tablets, 7.5 mg, once/day + Indomethacin Cuptalam Plaster, twice/day, 12 h at a time	3	VAS pain TER	-1.3↓ Treatment effect:↑ 100% vs. 91.83%	< 0.01 < 0.05

*a N* Number of patients included, *b* Age reported in years as a mean, *c CPM* with the Chinese herbs have potential benefits in chronic musculoskeletal pain relief and physical function, *VAS* pain(range 0–10), *WOMAC* pain (range 0–20). A lower score indicates a better outcome (less pain or better physical function). *TER* Total effectiveness rate, range 0–100%, higher score = better outcome. Four topical CPM including Xiaotongtie plaster, Fufang Nanxing Zhitong plaster, Tongluo Qutong plaster, and Qingpeng Ointment

Analgesic effects: Aconite Root, Radix Angelicae dahuricae, Pricklyash Peel, Kadsura Pepper Stem, Manchurian Wildgniger. Anti-inflammatory and healing effects: Angelicae Sinensis Radix, Curcumaelongae Rhizoma, Safflower, Szechuan Lovage Rhizome, Olibanun, Myrrha

### Trial sequential analysis

Multiple updates of the literature in systematic evaluations inevitably involve the risk of random errors and false positives [13]. Therefore, we can estimate and correct potential random errors, reduce the risk of false positives, and estimate the robustness and reliability of the system evaluation findings with the help of test sequential analysis (TSA) [14]. Also, based on the adequate estimation of type I errors as well as type II errors, TSA software can further calculate the minimum sample size for maximum confidence, which is the required information size (RIS).In the graphs drawn by TSA software, when the Z-curve crosses the traditional boundaries as well as the TSA boundaries or directly crosses the RIS, the conclusions of the analysis are sufficiently stable and reliable that further studies are unlikely to reverse the conclusions, and when the Z-curve crosses the invalid line into the invalid zone, we consider that there is no statistical difference between topical Chinese medicines for chronic musculoskeletal pain, and if the Z-curve does not meet the above 2 lines, it means that further clinical studies need to be developed to clarify the effectiveness of topical Chinese medicines “28”. TSA software (http://www.ctu.dk/tsa) was used for all these analyses.

## Results

**Fig. 2:**
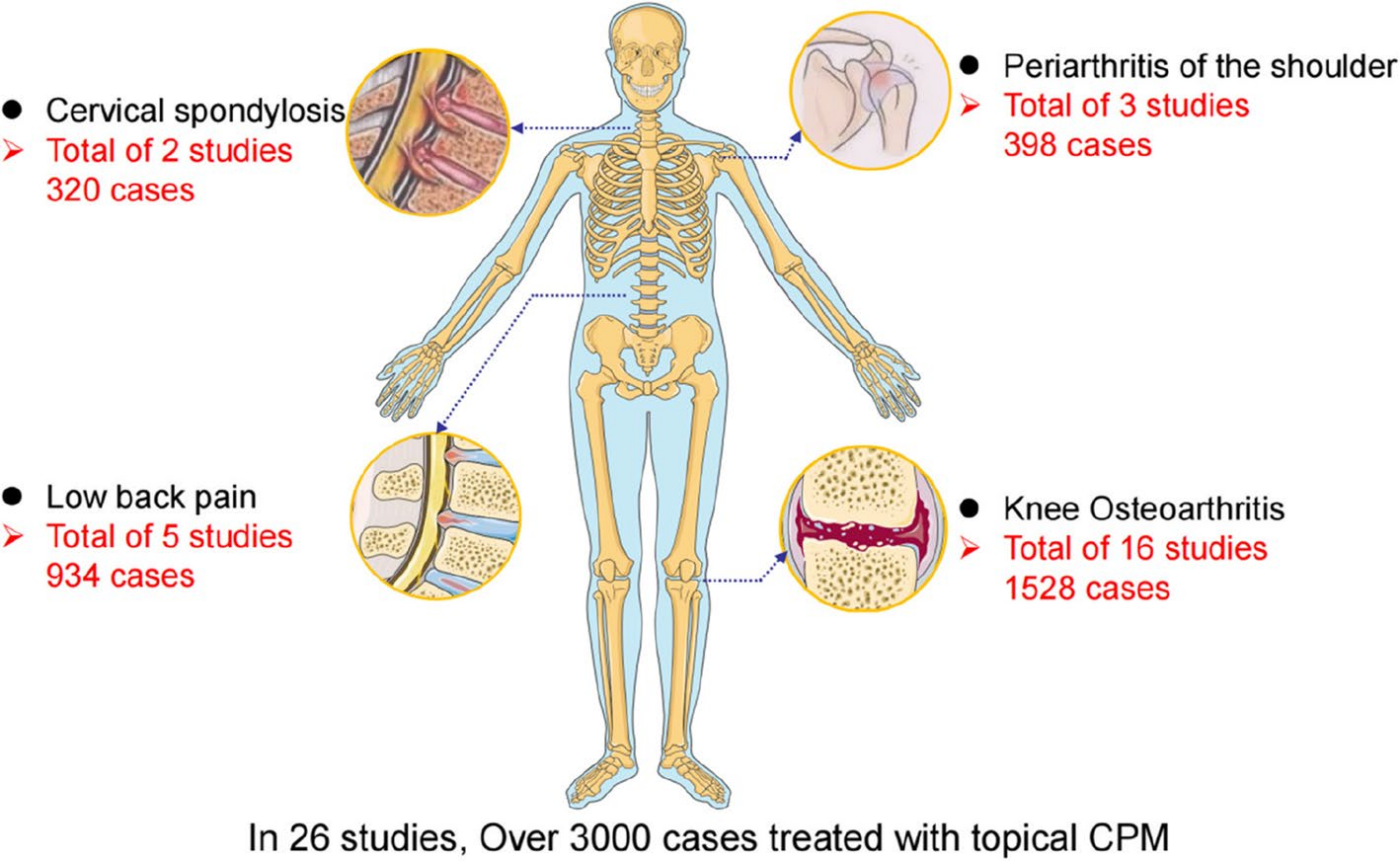
Summary of the number of cases in the literature of topical Chinese patent medicine for chronic Musculoskeletal pain

Through literature search, 2980 articles were initially searched and 2012 articles were obtained after removing duplicates. 120 articles were obtained after excluding 1892 articles again by reading their titles and abstracts, and then 87 articles were excluded after reading the full text of the articles for those that were not randomised controlled trials, missing outcome indicators, and incomplete data. Twenty-six RCTs with a total of 3180 participants met eligibility criteria, all published between 2010–2021, including 16 articles on knee OA, 2 studies on cervical spondylosis, 5 studies on lower back pain and 3 studies on frozen shoulder (Table 1 and Fig. 2). At the same time, we also searched the systematic evaluation and meta-analysis to verify our results, and obtained 6 relevant articles (Table 2).

**Table 2:** Summary reviews of topical Chinese patent medicine for chronic musculoskeletal pain

Authors, year	Type of study	Number of included studies	Comparison of interventions	Conclusion
Cai, 2020 [22]	systemic review meta-analysis	9 RCTs for knee OA	Fufang Nanxing Zhitong Plaster vs external application of a Western medicine analgesic ointment or placebo ointment, Oral NSAIDs	Fufang Nanxing Zhitong Plaster have higher clinical efficacy and can significantly improve pain and dysfunction, and they are comparable in terms of adverse reactions
Bai, 2020 [23]	systemic review meta-analysis	12 RCTs for knee OA	Xiaotongtie Plaster vs ntra-articular injection of sodium hyaluronate, oral glucosamine or oral painkillers	Xiaotongtie Plaster can significantly improve pain and has advantages in promoting the recovery of knee joint function as well as in improving swelling and tenderness
Sun, 2018 [24]	systemic review network meta-analysis	26 RCTs for knee OA	Xiaotongtie Plaster vs Diclofenac cream, diclofenac sodium tablets, sodium yaluronate, acupuncture therapy, glucosamine capsule, sodium vitonate + acupuncture therapy, sodium hyaluronate + Chinese medicine fumigation, sodium vitonate + betamethasone, sodium vitonate + ozone, sodium vitonate + oral Chinese medicine	The network meta-analysis included 11 treatment measures, of which the Xiaotongtie Plaster was ranked second for significantly improving the symptoms of knee OA
Connie Chen, 2020 [58]	systemic review meta-analysis	22 RCTs for knee OA	Xiaotongtie Plaster vs Oral NSAIDs, glucosamine or intra-articular injections of corticosteroids and hyaluronic acid	Xiaotongtie Plaster can significantly improve pain and joint stiffness and restore joint function
Yang, 2020 [25]	systemic review meta-analysis	5 RCTs for soft-tissue injury	Xiaotongtie Plaster vs Oral NSAIDs, muscle relaxants or physical therapy	Xiaotongtie Plaster may have advantages in improving pain, swelling, tenderness and dysfunction, and no serious adverse reactions have been found. Xiaotongtie Plaster may have advantages in the treatment of soft tissue injuries
Yang,M, 2021 [28]	systemic review meta-analysis	9 RCTs for LBP	Xiaotongtie Plaster vs Oral NSAIDs, physical therapy such as ultrashort waves	Xiaotongtie Plaster significantly improved pain and improved lumbar spine function, which was closely related to the improved long-term prognosis of LBP, and showed no difference in adverse effects between the two groups

*RCTs* Randomized controlled trials, *OA* Osteoarthritis, *NSAIDs* Non-steroidal anti-inflammatory drugs, *LBP* Low back pain

**Fig. 3:**
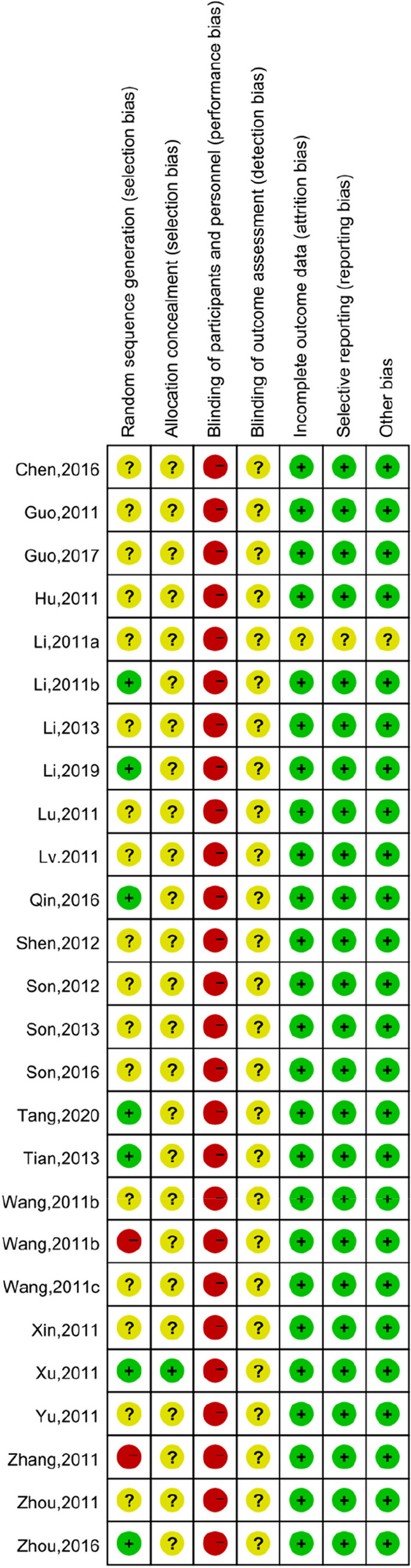
Risk of bias distribution graph

**Fig. 4:**
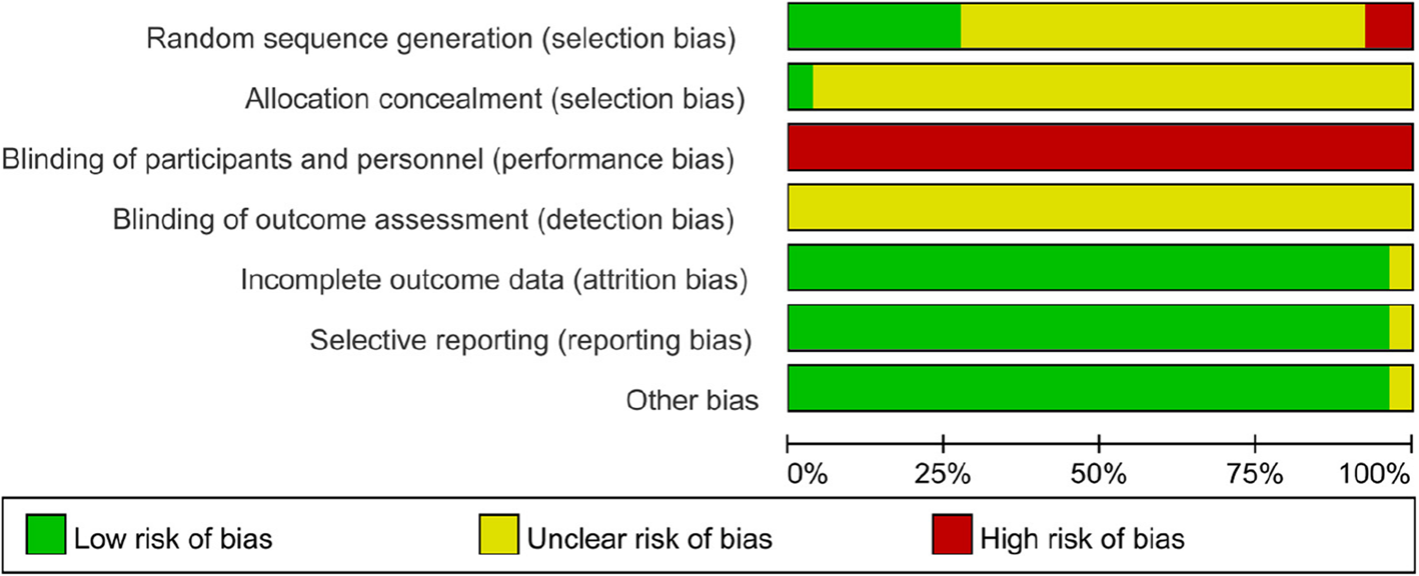
Cochrane risk of bias assessment

Figure 3 and Fig. 4 summarize the literature quality according to the Cochrane Risk of Bias Assessment tool. Table 1 summarizes the evidence reviewed according to the literature. Table 2 summarizes the characteristics of all systematic reviews and meta-analyses related to different types of CMP. Table 3 summarizes the evidence and main impacts of topical CPM for CMP.

**Table 3:** Summary of evidence on the efficacy of topical Chinese Patent Medicine in the treatment of chronic musculoskeletal pain

Interventions	Clinical domains and number of studies			
	">VAS pain (***n*** = 15)	WOMAC pain (***n*** = 1)	Total effectiveness rate (***n*** = 15)	
Xiaotongtie plaster	7 +	1 +	10 +	2-
Fufang Nanxing Zhitong plaster	6 +		1 +	1-
Tongluo Qutong plaster	1 +		1+	
Qingpeng Ointment	1 +			

*n* Number of studies

+ Overall beneficial effect, Lack of definite therapeutic effect, Blank, – No study

### Topical CPM and chronic joint and musculo-soft tissue pain

In this study, 19 RCTs in the literature were searched for chronic joint and soft-tissue derived pain: 16 RCTs in knee OA and 3 RCTs in Periarthritis of the shoulder. All of the RCTs were conducted in China and published between 2010 and 2021 (Table 1). Of these 19 clinical trials, 14 used Xiaotongtie Plaster, 3 studies of each used Fufang Nanxing Zhitong Plaster, 2 studies used Tongluo Qutong Plaster.As controls, 11 trials used oral NSAIDs (celecoxib capsules, diclofenac tablets and meloxicam tablets), 5 used topical NSAIDs (topical voltaren emulsion and indomethacin cuptalam plaster), 2 used physiotherapy and functional exercise and 2 Articular injections. Specifically, of the 19 studies on arthritogenic pain, 10 described a increase in total effectiveness rate, 8 described a decrease in VAS pain scores, 1 described a decrease in WOMAC [15–21]. In terms of the total effective rate, the average effective rate of Topical Chinese Patent Medicine was found to be 90.82%, which was higher than the 75.80% observed in the control group.Regarding the pain scores, a -1.30-point difference was observed between the VAS score of the treatment group and the control group, and a -0.82-point difference was observed between the WOMAC score of the treatment group and the control group.

On 3 RCTs to treat periarthritis of shoulder, 3 of them reported better clinical response effectiveness (average value 90.29% vs 78.87%). And one of these RCTs described the scores by VAS simultaneously (-1.3).

In the past 10 years, a total of five systematic reviews and meta-analysis (3 KOA, 2 muscle soft tissue), control treatment measures mainly include oral or topical NSAIDs, intra-articular injection of sodium vitonate, physical therapy and placebo, have reached the consistent conclusion that local CPM clinical efficiency, can significantly improve pain and dysfunction(mean difference: -1.78, 95% confidence interval [-2.58, -0.98], *P* < 0.0001 and mean difference: -1.84, 95% confidence interval [-2.42, -1.26], *P* < 0.0001), and in adverse effects with Western medical treatment, both were comparable [22, 23]. Another latest network meta-analysis of the efficacy of applying topical CPM was equally optimal for improving clinical symptoms of knee OA, also supporting previous studies on [24]. For the musculo-soft tissue pain, the topical CPM improved the pain more significantly (*P* < 0.05), but there was no difference in the relief of functional impairment (*P* > 0.05) in [25].

### Topical CPM and spine-derived pain

7 RCTs conducted in the past 10 years on topical CPM for spine-derived pain were included: 5 for LBP, 2 for cervical spondylosis. Of these 7 studies, 3 used physical therapy, 2 used oral pain medication (diclofenac sodium tablets), and one each used voltaren emulsion, capsaicin cream, and functional exercise. Compared with these therapeutic measures, 7 studies concluded that topical CPM was superior in its ability to relieve spine-derived pain, the mean VAS relief score in the topical CPM group versus the control group was -1.70. And 3 studies concluded that topical CPM had higher clinical response efficiency(average value 90.29% vs 78.87%).

In patients with LBP, topical CPM provided rapid relief of pain and dysfunction and reduced clinical symptoms in patients with acute LBP compared to conventional analgesics [26], but for more severe pain, relatively small changes in the magnitude of effects on pain and function were found (see Table 1).

In the past 10 years, one systematic review summarized 9 higher-quality RCTs evaluating the efficacy and safety of topical CPM in 1674 patients with LBP and found positive effects of topical CPM for chronic LBP (mean difference of 0.72, 95% confidence interval [0.49, 1.05], *P* < 0.0001) (see Table 2).

### Adverse reactions

The adverse reactions reported between studies mainly included skin flushing and itching, with a total of 10 cases of mild-to-moderate skin irritation reported in patients with topical CPM, while 11 cases of mild-to-moderate skin irritation occurred with conventional Western medicine. Thus, the two treatment modalities were comparable in terms of adverse reaction rates. In patients with spinal-origin pain, according to the literature of our included studies as well as the latest systematic review in 2021, a total of 64 patients using topical CPM presented with localized pruritus, rash, and blistering of the skin in the neck or lumbar region, necessitating clinical attention.

### Trial sequential analysis (TSA)

**Fig. 5:**
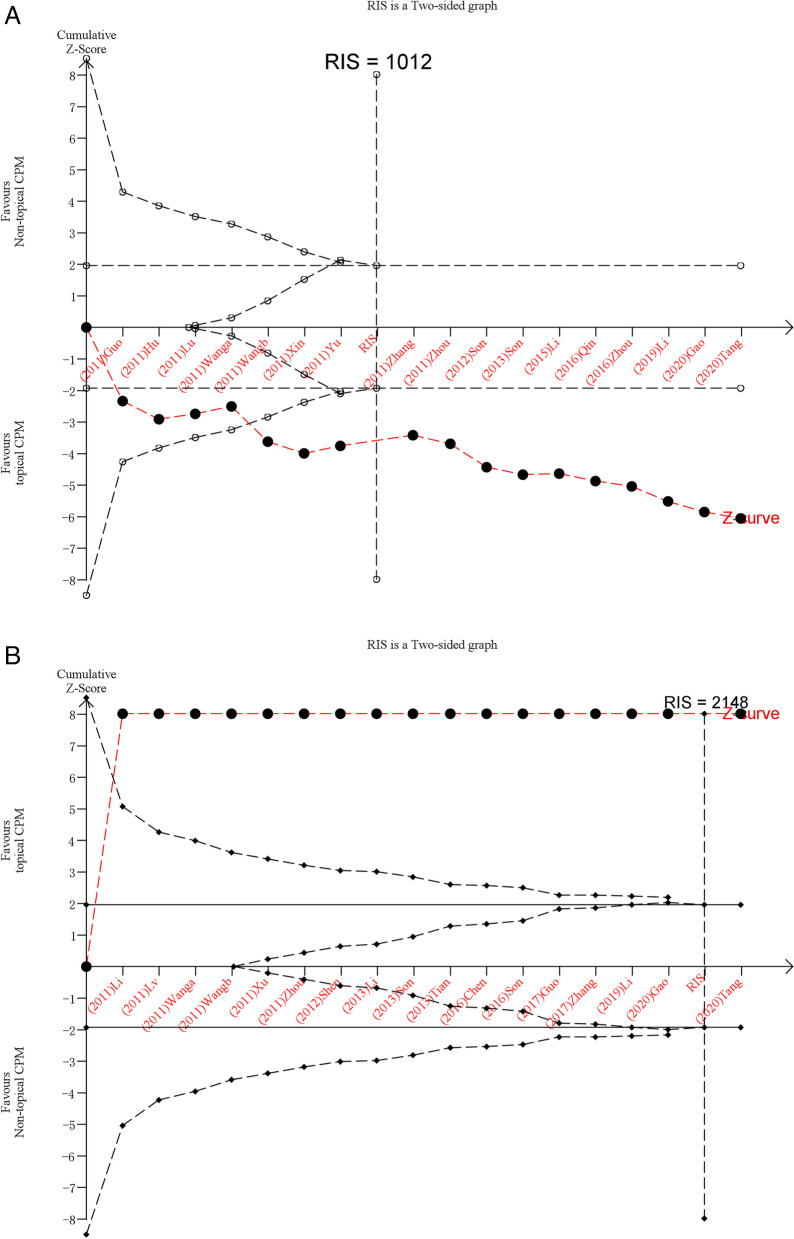
Trial sequential analyses of VAS pain score (**A**) and total effectiveness rate (**B**)

To minimize false positive results caused by random errors, analysis of the vas pain score and total effectiveness rate was performed using TSA software. As shown in Fig. 5, the cumulative Z-curves on vas pain score and total effectiveness rate cross the traditional significance and TSA bounds and the RIS boundary (1962 for vas pain score and 1405 for total effectiveness rate), indicating that the currently available evidence is largely conclusive and further studies cannot reverse this result.

## Discussion

To our knowledge, this is the first systematic review of topical CPM for CMP. By summarizing and arranging the evidence over the last decade, our study suggests that topical CPM may play a positive role in pain relief for CMP and appears to have a certain therapeutic effect on the dysfunction caused by CMP.

The studies included in this review have shown that topical CPM has significant analgesic effects on CMP, and some studies have also established that these effects are even more effective than those of oral or topical NSAIDs in the control group. A total of 26 articles involving 3180 patients were included in this study, of which 15 articles, involving 1567 patients, were within 2 weeks of treatment, indicating that the analgesic effect of topical CPM was superior to that of the control group in the short-term 2-week period, suggesting that topical CPM is undoubtedly more beneficial for patients in the acute phase [16].

In terms of *Topical CPM and chronic joint and musculo-soft tissue pain*, the evidence to date may tend to suggest that the once-daily application of topical CPM has certain advantages in improving the pain and clinical symptoms of joint and muscle-soft tissue-derived pain and clinical symptoms, and is more beneficial than traditional Western medication or physical therapy. In a study conducted in 2020, topical CPM resulted in significant improvement in muscle strength in the shoulder joint [21]. In general, topical proprietary Chinese medicines are primarily effective in relieving pain and possess some efficacy in restoring joint function. However, there is a paucity of research on the joint function part, which requires further exploration. In *Topical CPM and spine-derived pain,* the current evidence supports the positive effect of local CPM on chronic spinal source pain. In a clinical study conducted in 2005 that included 737 cases of cervical spondylosis, topical diclofenac sodium emulsion was used as a control, and topical CPM (Xiaotongtie Plaster) was found to provide significant pain relief from the initial application and sustained pain relief after five days of use, as well as beneficial effects on functional impairment [27]. In addition, some evidence suggests that the cost-effectiveness advantage of topical CPM for the treatment of patients with chronic LBP is outstanding [29]. But the pain improvement in patients with severe pain is still insufficient reported in some studies, so a combination of multiple treatments, such as combining oral NSAIDs, needs to be considered.

Different results for patients with functional impairment seem to be given by different studies when topical CPM is used to treat patients with CMP. There appears to be no significant difference in the study by Zhou et al. [27]. We think that may be caused by the different diseased body parts of different patients, as the mechanism of dysfunction and the degree of dysfunction are too dissimilar.

### Chemical composition and pharmacological research

Due to the controllability of the dose and composition of topical CPM and the convenience of clinical use, the topical application of topical CPM is not limited to a single efficacy study. Taking Xiaotongtie Plaster as an example, it is made of *Lamiophlomis rotata, thorn bean, turmeric, prickly ash, and buffalo horn,* and the main chemical constituents reported thus far are *cyclic ether terpenes, flavonoids, volatile oils*, etc. [28]. Studies have confirmed that iridoid glycosides can significantly increase the hot-plate pain threshold in mice, reduce the amount of writhing, significantly inhibit the swelling of the feet and ears of rats caused by xylene, and exert good anti-inflammatory and analgesic effects [29]. Xiaotongtie Plaster can exert an anti-inflammatory effect by inhibiting the *NF-κB* signaling pathway, reducing inflammatory cytokines (*TNF-α* and *IL-1β*), *COX-2* and its metabolite *PEG2* [30], and research also suggests that Xiaotongtie Plaster inhibits the production of *leukotriene B4* as well as the 5-lipoxygenase pathway, which may be another mechanism of its anti-inflammatory effect. In addition, Xiaotongtie Plaster can also reduce the blood flow rate and promote edema absorption to improve closed soft tissue injury [31]. Fufang Nanxing Zhitong Plaster mainly includes *Arranthaceae, Chuanwu, clove, cinnamon, camphor, borneol,* and other multiflavored medicinal materials, and its chemical constituents include *alkaloids, flavonoids, coumarins, organic acids, and chuanxiong lactones* [32]. Pharmacological studies have shown that Fufang Nanxing Zhitong Plaster can reduce the oxidative stress in the joints, thereby decreasing the degree of pain [33]. At the same time, studies have found that Fufang Nanxing Zhitong Plaster can increase the content of *β-endorphin* in rats, thereby improving the pain threshold of swelling and reducing the amount of writhing in mice induced by acetic acid, which has a certain analgesic effect [34].

Overall, topical CPM is administered percutaneously and usually contains volatile components, which makes the ointment have strong permeability and act directly and effectively on the diseased tissue, as well as allows the drug to enter the systemic circulation at a constant speed and avoid the liver first-pass effect, improves bioavailability, and reduces adverse reactions [35]. Pharmacological studies have shown that local application of topical CPM can improve the blood flow rate in the microcirculation, accelerate blood circulation, improve the microcirculation disorder in local inflammation, reduce its exudation, and lessen local inflammation or the oxidative stress response [36]. Although there have been some conjectures on the mechanism of external application of topical CPM for CMP, there are multiple kinds of topical CPM in China, many of which are only in the clinical trial stage, and the ingredients of the various Chinese medicines are different; consequently, the mechanism of analgesia and tissue repair still needs more research to provide evidence. Indeed, further high-level studies are needed to confirm this mechanism [37].

### Meaning and limitations

Patients with CMP usually have primary diseases. In addition to taking primary drugs, too many oral drugs are not only likely to cause more complicated adverse reactions but are also not conducive to patient compliance with treatment. Therefore, topical drug therapy plays a role that should not be ignored. Because the efficacy of TCM is being gradually recognized and the official guidelines support the treatment of CMP with topical drugs, the clinical efficacy of topical CPM has also received growing attention, which has prompted an increasing number of clinical trials. Our study found that topical CPM plays a positive role in providing relief from CMP, has a certain therapeutic effect on the dysfunction caused by CMP, and has a very prominent analgesic advantage in the short term (7 days). These findings are important for clinical practice, as physicians can explore potential new approaches and rethink their strategies to provide additional, safer treatment options for patients with CMP. The benefits of these topical CPMs should be validated in future large, high-quality studies.

Our study not only covered RCTs but also included several systematic reviews, which basically covered the common primary diseases related to CMP. However, there are still some deficiencies in the research. First, there was a low quality of the included RCTs, and there were obvious methodological defects. Second, there are many types of topical CPMs for the clinical treatment of CMP, and we only included the 4 most frequently used ones. Finally, all the RCTs were conducted in China, indicating that the generalizability of the evidence is limited. Therefore, the potential benefit of such treatments needs to be further evaluated through higher-quality clinical trials in more countries.

## Conclusion

In conclusion, topical CPM may be a valuable treatment option for CMP with a positive effect on pain relief, especially in the short term (within two weeks). However, given the methodological shortcomings of the included studies, more well-designed and controlled RCTs are still needed to support the clinical application of this treatment regimen in patients with CMP.

### Supplementary Information

**Additional file 1. **Search strategy.

## Data Availability

All data analyzed or generated in the work are included in published articles.

## Abbreviations

CPM: Chinese patent medicine
CMP: Chronic musculoskeletal pain
OA: Osteoarthritis
LBP: Lower back pain
NP: Neck pain
FS: Periarthritis of the shoulder
TCM: Traditional Chinese medicine
TER: Total effectiveness rate
TSA: Trial sequential Analysis
RCTs: Randomized controlled trial
NSAIDs: Nonsteroidal anti-inflammatory drugs
VAS: Visual analog scale
WOMAC: Western ontario and mcmaster universities arthritis index

## References

[1] Treede RD, Rief W, Barke A (2015). A classification of chronic pain for ICD-11[J]. Pain.

[2] Nicholas M, Vlaeyen J, Rief W (2019). The IASP classification of chronic pain for ICD-11: chronic primary pain[J]. Pain.

[3] Perrot S, Cohen M, Barke A, et al. The IASP classification of chronic pain for ICD-11: chronic secondary musculoskeletal pain[J]. Pain. 2019;160(1):77–82. 10.1097/j.pain.0000000000001389 30586074

[4] Cao BX, Lin XQ, Wu Y (2021). Chronic pain classification catalogue and definitions[J]. C J Pain Med.

[5] Briggs AM, Woolf AD, Dreinhofer K (2018). Reducing the global burden of musculoskeletal conditions[J]. Bull World Health Organ.

[6] Bond M (2012). A decade of improvement in pain education and clinical practice in developing countries: IASP initiatives[J]. Br J Pain.

[7] Duran J, Zitko P, Barrios P (2021). Chronic musculoskeletal pain and chronic widespread pain in chile: prevalence study performed as part of the national health survey[J]. J Clin Rheumatol.

[8] Bannuru RR, Osani MC, Vaysbrot EE (2019). OARSI guidelines for the non-surgical management of knee, hip, and polyarticular osteoarthritis[J]. Osteoarthr Cartilage.

[9] Foster NE, Anema JR, Cherkin D (2018). Prevention and treatment of low back pain: evidence, challenges, and promising directions[J]. Lancet.

[10] Oltean H, Robbins C, van Tulder MW, et al. Herbal medicine for lowback pain[J]. Cochrane Database Syst Rev. 2014(12). 10.1002/14651858.CD004504.pub4. 10.1002/14651858.CD004504.pub4 PMC7197042 25536022

[11] The Joint Surgery Branch of the Chinese Orthopaedic Association, The Subspecialty Group of Osteoarthritis,Chinese Association of Orthopaedic Surgeons, The National Clinical Research Center for Geriatric Disorders (Xiangya Hospital), et al. Chinese guideline for diagnosis and treatment of osteoarthritis (2021 edition) [J]. Chinese J Orthop. 2021:41(18):1291–314. 10.3969/j.issn.1006-9852.2018.12.001.

[12] Zheng XY (2002). Guidelines for Clinical Research of New Traditional Chinese Medicine (Trial) [M].

[13] Brok J, Thorlund K, Wetterslev J (2009). Apparently conclusive meta-analyses may be inconclusive–Trial sequential analysis adjustment of random error risk due to repetitive testing of accumulating data in apparently conclusive neonatal meta-analyses[J]. Int J Epidemiol.

[14] Wetterslev J, Thorlund K, Brok J (2008). Trial sequential analysis may establish when firm evidence is reached in cumulative meta-analysis[J]. J Clin Epidemiol.

[15] Guo PL, Xu YS, Ma YF (2011). Effect of Qizheng Pain-relieving paste on osteoarthritis. China Medical Herald.

[16] Xu K, Gao HY, Jiang Y (2011). Clinical study on the treatment of knee osteoarthritis with Xiaotongtie Plaster. Contemporary Medicine.

[17] Xin L (2011). Qi-Zheng Xiaotong Tiegao plaster in treatment of knee osteoarthritis. J Clinic Experiment Medicine.

[18] Shen HR, Zhao JS, Wang GY (2012). Clinical observation of the compoud Nanxing analgesic ointment for treating cold-dampness numbness type knee osteoanthritis. Chinese J Modern Drug Applic.

[19] Son PF, Kan WB, Jiang YX (2012). Clinical observation of “Fufang Nanxing Paste” in treating knee osteoarthritis. Shanghai J Tradition Chinese Medicine.

[20] Li X, Xu YQ (2015). Observation on curative effect of "Skilled Hands" Zhentong Huoluo tincture on knee osteoarthritis. Medic J National Defend Forces Southwest China.

[21] Qin YM (2016). To Explore the Curative Effect of Treating Knee Joint Osteoarthritis with Rheumatism and Blood Stasis Syndrome by Dredging Collaterals and Re-moving Pain Plaster. China Foreign Medical Treatment.

[22] Tang ZX (2020). Clinical Study on Xiaotong Plaster Combined with Moxibustion for Scapulohumeral Periarthritis of Cold-Dampness Obstruction Type. J New Chinese Medic.

[23] Cai X, Tang F, Ma WK (2020). Meta-analysis of Therapeutic Effect of TCM Paste on Knee Osteoarthritis. Rheumatism and Arthritis.

[24] Bai X, Wen JM, Yang SH (2020). Systematic Evaluation on Clinical Efficacy and Safety of Qizheng Xiaotong Plaster for Treatment of Knee Osteoarthritis. Chinese Informa Tradition Chinese Medic.

[25] Sun LK, Zhang XJ, Liu ZY (2018). Evaluation the efficacy of 11 kinds of interventions in the treatment of knee osteoarthritis. Tianjin J Tradition Chinese Medic.

[26] Yang SH, Zhang Y, Lin XF (2020). Systematic review on clinical efficacy and safety of Cheezheng Pain Relieving Plaster for soft tissue injury. China J Chin Materia Med.

[27] Li BJ, Ding WY, Shen Y (2011). Long-term efficacy analysis of Qizhengxiaotongtie plaster combined with Qizhengqingpeng ointment in degenerative lumbar spine disease. Chinese J Clinic Rational Drug Use.

[28] Dong FH (2005). Clinical observation of Qizheng Xiaotong Plaster in the treatment of cervical spondylosis. Continuing Medical Education.

[29] Yang M, Li SQ, Smith CM (2021). Tibetan herbal pain-relieving plaster for low back pain: A systematic review and meta-analysis[J]. Biomed Pharmacother.

[30] Wang X, Pei B, Xie YM (2014). Pharmaco-economic Research of Qizheng Xiaotong Plaster for treatment of Acute and prolonged lumbar sprain. World Chinese Medicine.

[31] Zhou Q. Clinical Observation of Self-Made Anti-inflammatory and Analgesic Ointment Combined with Functional Exercise in the Treatment of Stage II Periarthritis of Shoulder (Cold Coagulation Type)[D]. Zhejiang University of Chinese Medicine; 2016.

[32] Fan PC, Ma HP, Hao Y (2016). A new anti-fibrinolytic hemostatic compound 8-O-acetyl shanzhiside methylester extracted from Lamiophlomis rotata[J]. J Ethnopharmacol.

[33] Zheng YM, Du WJ, Yin XF (2015). Comparison of anti-inflammatory and analgesic effects of different effective parts of Angelica sinensis. Lishizhen Medic Materia Medica Res.

[34] Peng SY, Liu Y, Bao XH (2011). Inhibition of 5-lipoxygenase and cyclooxygenase-2 pathways by pain-relieving plaster in macrophages[J]. Pharm Biol.

[35] Wang YZ, Guo CY, Zhong HG (2008). In vivo effects of Pain Relieving Plaster on closed soft tissue injury in rabbit ears[J]. BMC Complement Altern Med.

[36] Li GS, Ma Y, Geng T (2019). Rapid identification of chemical components in compound Nanxing acesodyne plaster by UPLC-Q-TOF-MS/MS. China J Chin Materia Med.

[37] Cao XH, Bai ZX, Sun CY (2020). Effects of external application of Compound Nanxing Zhitong ointment on joint function and oxidative stress indexes in patients with knee osteoarthritis. Global Tradition Chinese Medic.

[38] Hu C, Chen YM, Yin SM (2009). The Approaches on Analgesic Effect and Mechanisms Of Nanxingzhitong Plaster. J Nanjing University Tradition Chinese Medic (Natural Scie).

[39] Hu YH, She YM, Han LY (2017). Clinical application of transdermal patch of Chinese materia medica. Chinese Traditional and Herbal Drugs.

[40] Tian J, Duo JZM, Chen LJ (2020). Research progress of Xiaotong Plaster. Chinese Tradition Patent Medicine.

[41] Chinese Medical Doctor Association Pain Medicine Branch, National Clinical Key Specialty·Pain Medicine Department of Sino-Japanese Friendship Hospital, Beijing Pain Treatment Quality Control and Improvement Center. Expert Consensus on Drug Treatment of Chronic Musculoskeletal Pain (2018)[J]. Chinese J Pain Med. 2018;24(12):881–87. 10.3969/j.issn.1006-9852.2018.12.001.

[42] Hu YM (2011). Clinical observation of Xiaotong Plaster in the treatment of knee osteoarthritis. China Modern Medicine.

[43] Wang CS, Liu WB, Yang ZG (2011). Qi zheng xiaotong tiegao treatment of Knee Osteoarthritis. Chinese J Aesthetic Medicine.

[44] Zhang Y (2011). Clinical observation of Qizheng Xiaotong Plaster in the treatment of knee osteoarthritis. Medical Information.

[45] Lv S (2011). The efficacy of Xiaotongtie Plaster in the treatment of knee osteoarthritis. Contemporary Medicine.

[46] Lu T, Li W (2011). Observation of curative effects on Qizheng Relieving-pain Paste in the treatment of knee osteoarthritis. China Medical Herald.

[47] Li W, Lu T, Ren J (2011). Treatment and clinical effect of knee osteoarthritis pain. J Practical Orthopaedics.

[48] Wang FY (2011). Qizheng Xiaotongtie Plaster pain relief effect and safety analysis. Chinese J Clinic Rational Drug Use.

[49] Li XZ, Xue JH, Gu BL (2019). Clinical Effect of Qizheng Xiaotong Plaster Combined with Fumigation External Therapy on Knee Osteoarthritis. Liaoning J Tradition Chinese Medic.

[50] Li DB, Luo H (2013). Clinical study of sodium hyaluronate coordinated by compound Nanxing analgesic Paster for treating 25 cases of knee osteoarthritis. China Pharmaceuticals.

[51] Chen W, Zhang C (2016). Knee osteoarthritis with rheumatic syndrome and stasis treated by Tongluo Qutong Plaster. J Changchun University Tradition Chinese Medic.

[52] Song YX, Li XF (2016). Therapeutic effect of Compound Nanxing Zhitong ointment on cervical spondylosis of cervical type. J Clinic Medical Literature (ElectronicEdition).

[53] Guo LS (2017). Study on the efficacy of compound Nanxing pain relief cream in treating 240 cases of cervical cervical spondylosis. Chinese Health Nutrition.

[54] Zhou SB (2011). The efficacy of Qizheng Xiaotongtie Plaster in treating 50 cases of lumbar muscle strain. J China Traditional Chinese Medic Informat.

[55] Wang YM, Li YM, Liu NQ (2011). Clinical observation of Qizheng Xiaotong Plaster in the treatment of lumbar disc herniation. Modern J Integrated Traditional Chinese and Weatern Medicine.

[56] Song MK. Clinical Efficacy Observation of Compound Nanxing Analgesic Ointment in the Treatment of Lumbar Muscle Strain[D]. Hubei: Huazhong University of Science and Technology; 2013. 10.7666/d.D413175.

[57] Tian ZG, Zhu XD, Zhu LF (2013). Effects on compound Nanxing analgesic cream in the treatment of non-speciifc low back pain. Chinese Journal of Medical Guide.

[58] Yu ZQ, Gu FS, Wang AG (2011). The efficacy of Qizheng Xiaotongtie Plaster combined with Tui Na therapy in treating 120 cases of frozen shoulder. Jilin Journal of Traditional Chinese Medicine.

[59] Chen C, Li SQ, Bao T (2020). A systematic review of Cheezheng pain relieving plaster for musculoskeletal pain: implications for oncology research and practice[J]. Integr Cancer Ther.

