# Supplementary material for: Topical Chinese patent medicines for chronic musculoskeletal pain: systematic review and trial sequential analysis
Source: BMC Musculoskelet Disord. 2023 Dec 20;24:985. doi: 10.1186/s12891-023-07072-8 (PMC10734070; doi:10.1186/s12891-023-07072-8)
Supplement: Supplementary file 1 — Additional file 1. Search strategy. [file 12891_2023_7072_MOESM1_ESM.doc]

**Search strategy**

**Pubmed:**

(((("Osteoarthritis, Knee"[Mesh]) OR (Knee Osteoarthritides[Title/Abstract])) OR (Knee Osteoarthritis[Title/Abstract])) OR (Osteoarthritis of Knee[Title/Abstract]) OR (Osteoarthritis of the Knee[Title/Abstract]) OR (Lumbar Muscle Strain[Mesh]) OR (Disc Displacement, Intervertebral[Mesh]) OR (Intervertebral Disc Displacement[Title/Abstract]) OR (Intervertebral Disc Displacements[Title/Abstract]) OR (Disk Protrusion[Title/Abstract]) OR (Disk Protrusions[Title/Abstract]) OR (Protrusion, Disk[Title/Abstract]) OR (Protruded Disk[Title/Abstract]) OR (Disk, Protruded[Title/Abstract]) OR (Protruded Disks[Title/Abstract]) OR (Intervertebral Disk Displacement[Title/Abstract]) OR (Disk Displacement, Intervertebral[Title/Abstract]) OR (Intervertebral Disk Displacements[Title/Abstract]) OR (Disk, Herniated[Title/Abstract]) OR (Herniated Disks[Title/Abstract]) OR (Slipped Disk[Title/Abstract]) OR (Disk, Slipped[Title/Abstract]) OR (Slipped Disks[Title/Abstract]) OR (Disk Prolapse[Title/Abstract]) OR (Disk Prolapses[Title/Abstract]) OR (Intervertebral Disc Herniation[Title/Abstract]) OR (Herniation, Intervertebral Disc[Title/Abstract]) OR (Intervertebral Disc Herniations[Title/Abstract]) OR (Intervertebral Disk Herniation[Title/Abstract]) OR (Herniation, Intervertebral Disk[Title/Abstract]) OR (Intervertebral Disk Herniations[Title/Abstract]) OR (Disk Herniation[Title/Abstract]) OR (Disk Herniations[Title/Abstract]) OR (Herniation, Disk[Title/Abstract]) OR (Periarthritis of Shoulder[Mesh]) OR (Cervical Spondylosis[Mesh]) OR (Tendinitis[Mesh]) OR (Lateral Epicondylitis[Mesh]) OR (Lumbar Osteoarthritis[Mesh])) AND (("Qi Zhen Pain Relief Plaster"[Title/Abstract]) OR ("Bone Pain Plaster"[Title/Abstract]) OR ("Compound Nanxing Pain Relief Ointment"[Title/Abstract]) OR ("Pain Relief Plaster"[Title/Abstract]) OR ("Tongluo Pain Relief Ointment"[Title/Abstract]) OR ("Yunnan Baiyao Ointment"[Title/Abstract]) OR ("Musk Strengthening Bone Ointment"[Title/Abstract]) OR ("Dog Skin Ointment"[Title/Abstract]) OR ("Qianshan Activating Blood Ointment"[Title/Abstract]) OR ("Injury and Dampness Pain Relief Ointment"[Title/Abstract]) OR ("Tianhe Bone Pain Plaster Ointment"[Title/Abstract]) OR ("Hu Li San"[Title/Abstract]))))

**Chinese National Knowledge Infrastructure (CNKI):**

(Title:(Qizheng Xiaotong Plaster) or Title:(Bone Pain Plaster) or Title:(Compound Nanxing Analgesic Ointment) or Title:(Xiaotong Plaster) or Title:(Tongluo Qu Pain Ointment) or Title:(Yunnan Baiyao Ointment) or Title:(Musk Strong Bone Ointment) or Title:(Dog Skin Plaster) or Title:(Qianshan Active Blood Ointment) or Title:(Injury Moisture Stop Pain Plaster) or Title:(Tianhe Bone Pain Plaster) or Title:(Hulisan)) and (Title:(Arthritis) or Title:(Ankle) or Title:(Shoulder) or Title:(Wrist) or Title:(Hand) or Title:(Finger) or Title:(Toe) or Title:(Elbow) or Title:(Hip) or Title:(Lumbar Muscle Strain) or Title:(Lumbar Disc Herniation) or Title:(Lumbar Spondylosis) or Title:(Cervical Spondylosis) or Title:(Rheumatoid Arthritis) or Title:(Periarthritis of Shoulder) or Title:(Rotator Cuff Injury) or Title:(Carpal Tunnel Syndrome) or Title:(Tenosynovitis) or Title:(Lateral Epicondylitis of Humerus) or Title:(Ankle Sprain) and Date:2010-*

**Wanfang data：**

(Title:("Osteoarthritis of Knee" or "Knee Osteoarthritis" or "Knee Degenerative Disease" or "Knee Joint Degenerative Changes" or "Knee Arthritis") and Title:("Diclofenac Sodium" or "Ketoprofen" or "Flurbiprofen" or "Ibuprofen" or "Lornoxicam" or "Topical Nonsteroidal Anti-inflammatory Drugs") and Title:("Qizheng Xiaotong Plaster" or "Bone Pain Plaster" or "Compound Nanxing Analgesic Ointment" or "Xiaotong Plaster" or "Tongluo Qu Pain Ointment" or "Yunnan Baiyao Ointment" or "Musk Strong Bone Ointment" or "Dog Skin Plaster" or "Qianshan Active Blood Ointment" or "Injury Moisture Stop Pain Plaster" or "Tianhe Bone Pain Plaster" or "Hulisan")) and Date:2010-*
